# The role of contextual factors in avenues to recover from gambling disorder: a scoping review

**DOI:** 10.3389/fpsyg.2024.1247152

**Published:** 2024-02-12

**Authors:** Dagny Adriaenssen Johannessen, Stian Overå, Espen Ajo Arnevik

**Affiliations:** ^1^Department of Social Work, Child Welfare and Social Policy, OsloMet—Oslo Metropolitan University, Oslo, Norway; ^2^Blue Cross East, Oslo, Norway; ^3^Norwegian National Advisory Unit on Concurrent Substance Abuse and Mental Health Disorders, Innlandet Hospital Trust, Brumunddal, Norway; ^4^Section for Clinical Addiction Research, Division of Mental Health and Addiction, Oslo University Hospital, Oslo, Norway

**Keywords:** gambling disorder, treatment, recovery, context factors, scoping review

## Abstract

**Introduction:**

Recovery from complex conditions such as gambling disorders (GD) often entail considerable change and require a range of adaptable interventions in the health care system. Outcomes from such avenues to change are influenced by multifarious contextual factors, which are less frequently considered in treatment outcome studies. Accordingly, this scoping review aims to map the level of evidence and explore how contextual factors influence the provision and outcomes of GD interventions.

**Methods:**

A systematic search in selected health and social science research databases yielded a total of 2.464 unique references. The results were screened in three selection steps—titles (*n* = 2.464), abstracts (*n* = 284) and full-text (*n* = 104). The scoping approach was applied to provide a narrative account of the final included references (*n* = 34).

**Results and discussion:**

Findings suggest that the research on GD treatment is in the early stages of development. Additionally, studies on GD interventions are characterized by cultural biases (Region and ethnicity and Gender perspectives), while three key elements are described as successful avenues to recover from GD (Competence, Perception and Utilization). In line with these findings, proposals for future research and treatment designs are made.

## 1 Introduction

Behavioral addictions, such as gambling disorders (GDs), are considered complex conditions (Griffiths, 2005; Langham et al., 2015). Approximately 0.1–3% of the population across European countries and around 0.1–6% worldwide experienced harmful consequences from GDs in the past year (Calado and Griffiths, 2016; Health Survey for England 2018, 2019; Pallesen et al., 2023).

Regarding GDs as addictive disorders, the classification of GD in the Diagnostic and Statistical Manual of Mental Disorders (DSM) has undergone significant changes, marking a watershed event in 1980 (Rosenthal, 2020). With the publication of the DSM-III (APA, 1980), GD was first acknowledged as a psychiatric condition. Termed “Pathological Gambling,” it was categorized under “Disorders of Impulse Control Not Elsewhere Classified.” The subsequent edition, DSM-IV (APA, 1994), retained this classification, albeit with more refined diagnostic criteria. A major paradigm shift occurred in DSM-5 (APA, 2013), where GD was reclassified as a “Substance-Related and Addictive Disorder,” reflecting contemporary research that highlighted parallels in brain function, behavioral patterns, and treatment approaches between GD and substance use disorders (Abbott, 2020; Moreira et al., 2023). Similarly, in the International Classification of Diseases (ICD), GD’s recognition as a mental health condition has evolved (Attard-Mallia, 2023). Initially classified under “Habit and Impulse Disorders” in ICD-10 (WHO, 1992), it was repositioned in ICD-11 to “Disorders Due to Addictive Behaviors,” mirroring the DSM-5’s stance (WHO, 2019). This reclassification in both the DSM and ICD marks an important development in acknowledging GD as a complex and significant mental health challenge globally (Johnstone and Regan, 2020; Shaffer et al., 2020).

Gambling disorders are complex in the sense that individuals with the disorder experience negative consequences in multiple concurrent domains of everyday life (Griffiths, 2005; Langham et al., 2015). Negative consequences include psychological distress (Petry, 2005; Lorains et al., 2011; Dowling et al., 2015), interpersonal conflict (Downs and Woolrych, 2010; Goodwin et al., 2017; Roberts et al., 2018) and social consequences, such as financial and housing problems (Grant et al., 2010c; Langham et al., 2015; Sharman et al., 2016; Swanton and Gainsbury, 2020). Studies from Europe (Winkler et al., 2017; Rogers et al., 2019; Hofmarcher et al., 2020), Australia (Productivity Commission, 2001; Victorian Competition and Efficiency Commission, 2012), and the United States (Walker and Sobel, 2016) have also proposed that GD is a public health issue in terms societal costs. Additionally, GDs are associated with various risk factors that contribute to their complex nature, such as younger age, male gender, low socioeconomic status, traumatic experiences, childhood neglect, and exposure to gambling environments (Abbott et al., 2018).

Arguably, the prevalence of GDs worldwide is modest from a population perspective. However, one person’s GD affects several of their significant others in a negative way (Goodwin et al., 2017; Järvinen-Tassopoulos, 2020; Lind et al., 2022), and GD-related repercussions have considerable societal costs (Atherton and Beynon, 2018; Hofmarcher et al., 2020; Kristensen et al., 2022). Taking these aspects together, GDs represent a major concern for individuals, families, and society.

Overcoming GDs often entails considerable personal, relational, and social change (Ashford et al., 2019; Brown and Ashford, 2019). Findings from recent studies on GDs emphasized certain traits as contributing to reduced potency of treatment, including distorted cognition (Rash and Petry, 2014; Mansueto et al., 2019; Yücel et al., 2019), impaired decision-making (Goschke, 2014; Challet-Bouju et al., 2017), impulsiveness and emotional dysregulation (Yau and Potenza, 2015; Anderson et al., 2021; Vintró-Alcaraz et al., 2021). Such traits have been identified as core maintaining factors and described as commonalities across addictive behaviors (e.g., Shaffer et al., 2004, 2018; Griffiths, 2005; Grant et al., 2010b; Mansueto et al., 2019; Yücel et al., 2019).

Most people with addiction-related problems recover without formal support from the health care system (Slutske, 2006; Kelly et al., 2017). However, some seek formal help to overcome their GD, where those with serious issues are more likely to seek formal treatment than those with less severe problems (Wieczorek and Dąbrowska, 2018; Bijker et al., 2022). These courses of change usually require individual tailoring and diversity of interventions and treatment delivery in the health care system (Clark, 2013; Kim and Hodgins, 2018) and have previously been described as complex (Pawson et al., 2004; Clark, 2013; Wong et al., 2013). Outcomes from complex avenues to change are influenced by multifarious factors along the way (Bhaskar et al., 2018; Danermark et al., 2018), including those activated through specific health care interventions. Interventions described as *cognitive behavioral* (CB) represents the most prevalent approaches for the treatment of GD (Chebli et al., 2016; Challet-Bouju et al., 2017; Petry et al., 2017; Abbott, 2019). These approaches include interventions such as Motivational Interview (MI) (Miller and Rollnick, 2004) and Personalized Normative Feedback (PFI) (Marchica and Derevensky, 2016). Mindfulness-based interventions (Maynard et al., 2018) and pharmacotherapy are also common treatment interventions for GDs (Bartley and Bloch, 2013). These treatment approaches aim to target factors specifically associated with the maintaining of GDs. As exemplified by Yücel et al. (2019), mindfulness or goal-directed strategies intend to readjust maintaining habits and counter compulsion, while contingency management or MI aims to target GD-related reward ideation. Moreover, previous research indicated positive outcomes from treatments addressing specific GD-related factors, including cognitive flexibility, emotional regulation (Sancho et al., 2018), self-awareness and self-management strategies (Marchica and Derevensky, 2016). GD treatment also shows beneficial results related to reduced gambling frequency, expenditure (Yakovenko et al., 2015; Maynard et al., 2018; Peter et al., 2019), and gambling urges (Christensen, 2018).

Although the findings from studies on GD treatment represent important contributions regarding whether GD-specific interventions coincide with beneficial change in certain life domains, less is known about how change is realized. Pawson et al. (2004) described specific interventions in complex avenues of change as “complex systems thrust amidst complex systems.” Accordingly, contextual factors distinctive from the place in which the interventions occur play an essential role in the eventual outcome of complex treatment avenues (Pawson et al., 2004; Bhaskar et al., 2018; Danermark et al., 2018). Contextual factors, such as policies, social equality, healthcare capacity and capability, and local environment factors, determine various aspects of health. As exemplified in previous studies, these contextual factors are associated with health outcomes, including lifestyle diseases, mortality, mental and physical health status, substance use, and criminal behavior (Bleich et al., 2012; Ciccone et al., 2014; Oakes et al., 2015; Vella et al., 2023). Considering GD treatment specifically, influencing contextual factors include political governance (e.g., regulation and access to gambling and treatment services), welfare (e.g., socioeconomic factors, accessibility and quality of support services), social factors (e.g., personal finances, leisure activities, employment, and living conditions), and individual factors (e.g., predispositions) (Abbott et al., 2018). However, these factors are less frequently considered in treatment outcome studies (Pawson et al., 2005; Richardson et al., 2018).

Research on outcomes from GD treatment has evolved gradually since the 80s when CB first was established as “best practice” (Ladouceur et al., 1994; Blaszczynski and Silove, 1995). The potency of a treatment intervention for bringing about change in the real world is termed “treatment effectiveness” (Office of Technology Assessment, 1978). That is, the effectiveness of a treatment is the expectation of a benefit when the typical practitioner provides the treatment in a typical fashion to typical patients in typical clinical settings. If the effectiveness of a new treatment is small or suppressed by the influence of unexplored mediating factors, the treatment does not achieve the status “effective.” The effectiveness relies on high internal validity, which is why mediating or moderating contextual factors are studied. The evaluation guidelines of World Health Organization (WHO, 1975) for testing and development of drugs’ medical use are often adapted. Building on these guidelines, Robey and Schultz (1998) proposed a five-phase model for outcome research, where phase I focuses on researching the therapeutic effect and, if it is present, estimating its magnitude. Phase II studies typically explore the dimensions of therapeutic effects and prepare for conducting a clinical trial. In phase III, clinical trials are conducted. Large sample sizes and conservative tolerance for type I errors are applied. In phase IV, the context is explored, and field research is typical. The fundamental task is to assess the degree to which the effect is realized in day-to-day clinical practice and to what extent different approaches are needed to meet the variations in target populations or contextual factors. Phase V typically focuses on cost–benefit analyses. Still, GD treatment is in its early stages of development and there has been extensive change—societal and technological—since the search for effective GD treatment first commenced. Additionally, compared with substance-related addictions GDs were recently recognized as standalone addictive behavioral diagnoses (APA, 2013; WHO, 2019). As a result, research on the treatment interventions for GDs has developed and increased in recent years.

The primary aim of this review is to broaden the understanding of how specific intervention features catalyze change and to guide future research directions by mapping the current evidence level for GD treatments. Additionally, it seeks to elucidate the role of contextual factors in these complex change processes. To achieve this, the review addresses two critical research questions:

1. What is the status of evidence for GD treatment?
2. How do contextual factors influence the provision and outcomes of interventions targeting emotional regulation or impulsivity in people with GDs?

## 2 Materials and methods

The review used a scoping approach to provide a narrative account of the initial search results. Scoping is a valid approach to map the main sources of evidence and gain insight to overarching patterns in research fields (Arksey and O'Malley, 2005; Lockwood et al., 2019). The aims of this review were explored by illuminating links between contextual factors and specific GD interventions.

The planning, procedure, and reporting of findings from the present review align with PRISMA extension for scoping reviews (PRISMA-ScR) (Tricco et al., 2018). The review was registered *a priori* in the International Prospective Register of Systematic Reviews (PROSPERO registration code: 282609).^1^

### 2.1 Preliminary scoping

First, the Cochrane Database of Systematic Reviews (CDSR) and Epistemonikos were searched in August 2021 for prior published reviews, and PROSPERO was searched for ongoing reviews with similar overarching aims as the present review. At the time of the search, no such reviews were identified.

**Figure 1:**
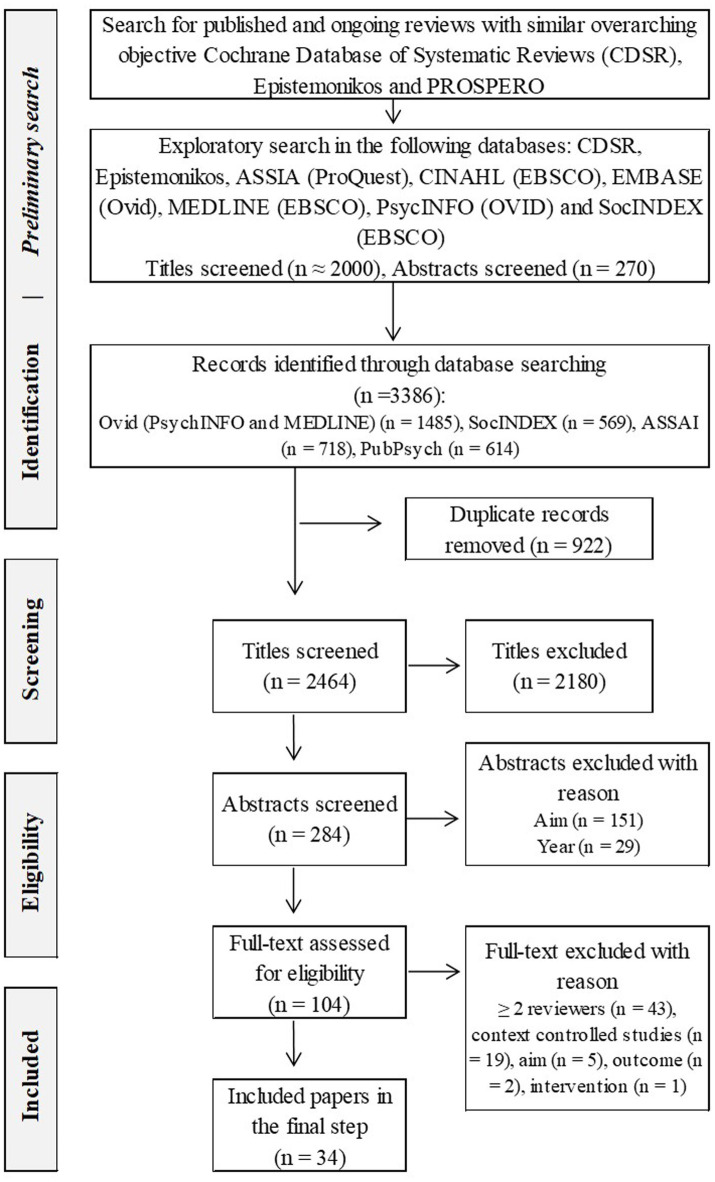
Screening and selection process.

As shown in Figure 1, the preliminary scoping was run in databases providing health and social science research: ASSIA (ProQuest), CINAHL (EBSCO), EMBASE (Ovid), MEDLINE (EBSCO), PsycINFO (OVID), and SocINDEX (EBSCO). The scope was conducted during September and October 2021 using a combination of relevant keywords: [(gambl* OR betti* OR wage*) AND (disorder* OR addict* OR dependen* OR excessive)) AND (treat* OR therap* OR rehab* OR recover* OR intervention*].

The search results were screened to identify the intended outcomes of prevailing GD interventions, focus the aims of this review and design a main search strategy. The results from the preliminary scoping were reviewed and discussed between all authors (DJ, SO, and EA), leading to an agreement to address the underlying traits that contribute to the maintenance of GDs. These traits include factors related to GD (e.g., cognitive flexibility, decision-making, impulsiveness, or emotional regulation) that prevailing interventions are designed to address and that are central to program theory. The main search (described in the next section) was adjusted according to findings from the preliminary search by adding keywords related to emotional regulation and impulsiveness to the search string.

### 2.2 Main search strategy

The main search was conducted in January and February 2022 in the following databases: ASSIA (ProQuest), MEDLINE (Ovid), PsycINFO (OVID), SocINDEX (EBSCO), and PubPsych (see Figure 1). The databases were selected to target the biopsychosocial aspects of GDs (WHO, 1992, 2019; APA, 2013).

The main search string was constructed by combining relevant keywords: (gambl*) AND (problem* OR disorder* OR addict*) AND (therap* OR treatment* OR self-help OR “user driven” OR “health care”) AND (urge* OR emotion* OR affect*). The search strategy was adapted to meet the terms given by each of the respective databases. The complete search strategy is presented in the Supplementary Information SI1.

#### 2.2.1 Inclusion criteria

The criteria of eligibility were informed by the preliminary scoping and aims of the present review. As outlined in Table 1, the inclusion criteria comprised *interventions targeting emotional regulation or impulsivity* (setting) *in people with gambling disorder* (population). The outcomes of interest were *the influence of contextual factors on the provision and outcomes of such interventions*.

**Table 1:** Inclusion criteria.

Phenomena of interest	Description
Population	People with gambling disorders or stakeholders engaged with health care services to promote recovery from gambling disorders (e.g., providers, peers, and clinicians).
	To gamble means to stake money or other values in the hope of profit, even if the betting outcome is unknown. Gambling disorder refers to dominant emotional, cognitive, and behavioral patterns.
Setting: (*intervention focus*)	Interventions aiming at promoting strategies to modify or replace an affective state and to identify the underlying cause of the affective state.^*^
Outcomes	Influential factors in the context surrounding the intervention or the recovery process, but which are unrelated to the specific intervention.
	Factors of interest are those that are unrelated to the specific intervention.

^*^Gross (2013).

Here, it is relevant to distinguish between context and setting. Context represents an element of the analytical framework and constitutes both observable and unobservable individual, intrapersonal, institutional and intra-structural surrounding traits (Pawson et al., 2004). The setting, on the other hand, refers to the characteristics of the intervention (Greenhalgh and Manzano, 2021). According to the inclusion criteria, interventions targeting emotional regulation or impulsivity were the setting of interest. Therefore, studies reporting the impact of interventions on individuals’ capacity to cope with overwhelming experiences and emotions were included during the screening process, while studies only reporting outcomes, such as gambling frequency or expenditure, were excluded. Also, studies written in other languages than English, studies designated to control for the influence of contextual factors [e.g., Randomized controlled trails (RCT)] and studies published before the year 2010 were excluded (see section 2.2.2 for description).

#### 2.2.2 Screening and selection process

The search was planned by all authors (DJ, SO, and EA) and conducted by DJ. As shown in Figure 1, the main search yielded a total of 3.386 references, including duplicates (*n* = 922). The results from the main search were screened using three selection steps: (1) titles, (2) abstracts, and (3) full-text publications. The screening and selection process was conducted using Bramer et al. (2017) procedure with EndNote 20.3.

In the first selection step, author DJ applied the inclusion criteria to titles (*n* = 2.464) and identified these as either *Include* or *Exclude*. A subset with a random selection of 20% was screened by the two co-authors to establish reliability.

**Figure 2:**
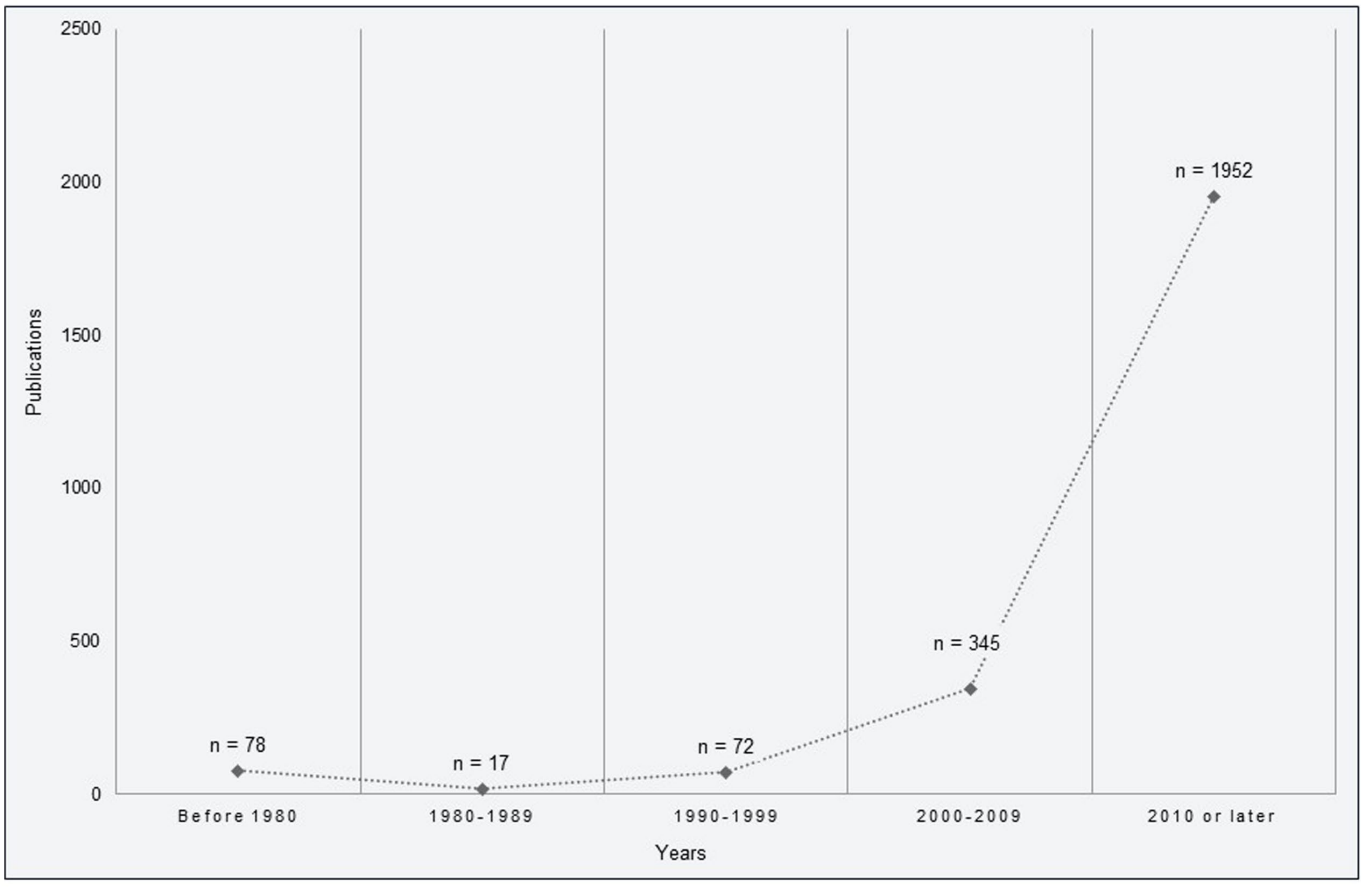
Publications per period.

In the second selection step, authors DJ, SO, and EA applied the inclusion criteria to evaluate references identified as *Include* (*n* = 284). Diverging evaluations were resolved by consensus. Based on the authors’ preliminary perceptions during the second selection step (abstracts), further directions of the screening process were discussed and decided on. Looking at the main search results (*n* = 2.464), most of the identified references were published in 2010 or later (1,952 publications vs. 512 publications before 2010; see Figure 2). Thus, studies published before 2010 and studies that only included diagnostic traits, such as time or money spent for gambling, were excluded. In addition, studies designed to control for the influence of contextual factors (i.e., RCTs) were excluded. These additional eligibility criteria were applied to further screening.

In the last selection step, all authors (DJ, SO, and EA) evaluated the included full-text publications (*n* = 104) and identified those as *Include* (*n* = 34) and *Exclude* (*n* = 70).

### 2.3 Information extraction

Relevant information was extracted from the finally included references and recorded in a predesigned data extraction form. In addition to the variables *author* and *year of publication*, information that was extracted focused on *study design*, *phenomena of interest* (e.g., study aims and objectives, operationalization’s, and definition of GD), *sample characteristics* (e.g., sample size, gender, age, ethnicity, and socio-demographics), *intervention* (e.g., type of therapeutic approach, context, and mode of delivery, intended outcomes), and *findings* and *explanation of findings*. The data extraction form is presented in the Supplementary Information SI2. Information extraction was carried out in two phases. First, DJ, SO, and EA extracted one-third of the information each from the finally included references. Second, DJ, SO, and EA reviewed the extracted information across the included references by each analyzing a set of variables.

#### 2.3.1 Considerations in summarizing the extracted information

The aims of the current review were to map the level of evidence and explore how contextual factors influence the provision and outcomes of interventions targeting emotional regulation or impulsivity in people with GDs. Information extracted from the finally included references (*n* = 34) was summarized with the purpose of providing a narrative account of findings. Following Arksey and O'Malley (2005), the narrative presentation of findings had two aspects: (1) a descriptive presentation of information extracted from the individual references, and (2) a presentation of patterns across the included references, specifically relevant to the aims of the present review.

Considering the aims, the contextual factors of interest are those present in the surroundings of the place in which GD interventions are provided. While the patterns of interest relate to contextual factors with influential potential on the provision or outcomes of GD interventions. The analyses of the initial search results therefore focused on processes or activities surrounding the specific GD interventions. Contextual factors refer to elements surrounding a health care intervention. As outlined by Pawson et al. (2004) contextual factors with influential potential include individual (i.e., characteristics of the actors that are involved, such as attitudes, expertise, and demographic traits), interpersonal (i.e., characteristics of the interaction between actors that are involved, such as communication, trust, and safety), institutional (i.e., characteristics of the local environment surrounding the change process, such as culture, management, and structure), and intra-structural factors (i.e., characteristics of the broader environment surrounding the change process, such as political or economic governance, welfare system, and regulation and organization of the health care system).

## 3 Results

### 3.1 Characteristics of the included studies

The sample sizes of the studies varied widely, with a range of 1–471 participants and a median of 27.5 (see Supplementary Table 1). Of the 34 intervention studies included in our scoping review, 23 were quantitative studies, eight were qualitative studies (case studies and interview studies), and three used mixed method designs. Of the 23 quantitative studies, 17 were categorized as exploratory studies with various pre–post outcome changes. Five studies had experimental designs, including one quasi-controlled study. According to the five-phase model proposed by Robey and Schultz (1998), most of the included studies of treatment interventions for GD fell into phase I or II in that they focused on therapeutic effect or dimensions of therapeutic effect. Here, three out of the 34 studies had medical interventions (Memantine; Grant et al., 2010a; Disulfiram; Mutschler et al., 2010; Naltrexone; Ward et al., 2018). The most reported psychotherapeutic interventions were cognitive behavioral therapy (CBT, *n* = 10: Castren et al., 2013; Rossini-Dib et al., 2015; Tarrega et al., 2015; Boughton et al., 2016; Smith et al., 2016, 2018; Bouchard et al., 2017; Mallorqui-Bague et al., 2018; Zhuang et al., 2018; Granero et al., 2020), which was delivered either in groups or individually, either face to face (F2F) or digitally. This was followed by motivational interviewing (MI, *n* = 4: Grant et al., 2011; Parhami et al., 2012; Pasche et al., 2013; Stewart et al., 2016), mindfulness based interventions (*n* = 3: de Lisle et al., 2011; Shead et al., 2020; van der Tempel et al., 2020), dialectical behavior therapy (DBT, *n* = 1: Christensen et al., 2013), acceptance and commitment therapy (ACT, *n* = 1: Nastally and Dixon, 2012), and transcranial magnetic stimulation (TMS, *n* = 1: Zack et al., 2016). Desensitization techniques or exposure were a part of multiple studies but were also used exclusively in two studies (Giroux et al., 2013; van Minnen et al., 2020). Several studies did not describe the specific content of the treatment (*n* = 9). Four of the interventions were delivered digitally, and the rest were delivered via F2F methods.

### 3.2 Cultural biases

A key finding addresses cultural biases with generative potential for the access to knowledge about people with GD. Considering the aims of this review, the cultural biases constitute the influencing contextual factor, while access to culturally adapted knowledge and interventions comprise the outcome of GD interventions.

We identified two cultural biases in the selected studies related to *Region and ethnicity* and *Gender perspectives*. The first bias, *Region and ethnicity*, related to the lack of studies examining non-Western societies and cultural subgroups (e.g., ethnicity, religion, and Indigenous peoples). Three regions were overrepresented in the 34 included studies (see Supplementary Table 1): North America (*n* = 14), Europe (*n* = 10), and Australia (*n* = 7). Most studies were conducted in Canada (*n* = 10), Australia (*n* = 7), Spain (*n* = 5), and the United States (*n* = 4). In total, three studies were from countries in South America (Brazil), Africa (South Africa), and Asia (China/Hong Kong).

Many studies (*n* = 19) provided no information about the ethnic composition of the sample. In two studies conducted in Canada, the sample consisted solely of Caucasian males (Stewart et al., 2016) and Caucasian women (Piquette and Norman, 2013). None of these studies included cultural factors in the analyses. Several studies included different ethnic groups (Grant et al., 2011; Christensen et al., 2013; Gomes and Pascual-Leone, 2015; Rossini-Dib et al., 2015; Tarrega et al., 2015; Boughton et al., 2016; Bouchard et al., 2017; van Minnen et al., 2020). However, none of these studies addressed cultural background in the analysis of the results.

We found that cultural factors were given attention in two of the three studies conducted in non-Western countries (Pasche et al., 2013; Zhuang et al., 2018) and in a study that specifically addressed an ethnic minority group (Parhami et al., 2012). A common feature of these studies is that they examined culturally adapted programs. For example, Parhami et al. (2012) investigated the effects of an intervention specifically designed for Chinese immigrants in the United States. However, there were no cross-cultural studies or studies about indigenous peoples in the sample.^2^

The second bias, *Gender perspectives*, points to a lack of research examining the impact of gender in the intervention of gambling problems. Most of the studies had samples of both males and females (*n* = 21). The majority of the gender-mixed studies had more male than female participants (*n* = 15); five studies had more female than male participants; and one study (Smith et al., 2016) had an equal gender composition. The sample size in these studies ranged from 3 to 471 participants, with an average of 43.5. Finally, one of the articles in the sample (Smith et al., 2018) presented results from three sub studies, two of which had approximately the same gender composition and with the last consisting of only male participants. The majority of the gender-mixed studies did not examine gender differences in the analysis, with Castren et al. (2013) and Rodda et al. (2017) as the only exceptions.

Seven studies used an all-male sample (Tarrega et al., 2015; Stewart et al., 2016; Zack et al., 2016; Mallorqui-Bague et al., 2018; Zhuang et al., 2018; Granero et al., 2020; Melero Ventola et al., 2020), and three studies used an all-female sample (Piquette and Norman, 2013; Boughton et al., 2016; van der Tempel et al., 2020). In the gender-homogeneous studies, the number ranged from 1 to 192 participants, with an average of 42.6. Further, there were two single case studies, one of which involved a 48-year-old man (Mutschler et al., 2010) and one involving a 61-year-old woman (de Lisle et al., 2011).

The gender perspective was not present in any of the studies that exclusively used male samples. In comparison, two of the three studies with only female participants (Piquette and Norman, 2013; van der Tempel et al., 2020) discussed gambling behavior in relation to gender roles in society.

### 3.3 Three elements in avenues of recovery

An additional key finding addresses the commonalities across successful avenues for change. This finding presents contextual factors with generative potential for the provision of GD interventions. Considering the aims of this review, the structure and organization of health care services constitute the influencing contextual factor, while the three elements represent provision of GD interventions.

Across the included studies, certain features were echoed in the treatment courses identified as *successful*. These can be summarized in three overarching elements with relevance to recovery from GD: *Competence*, *Perception*, and *Utilization*. The element *Competence* encompasses insight, awareness or knowledge related to recovery from GD (e.g., understanding emotions, cognition, behavior, coping strategies, literacy, GD-specific factors, and the extent of available help or services). Examples from the included studies contain sharing experience with peers to gain knowledge about GD and learn efficient GD-specific coping strategies (e.g., Piquette and Norman, 2013; Boughton et al., 2016; Syvertsen et al., 2020). Additionally, cognitive flexibility (e.g., Grant et al., 2011; Mallorqui-Bague et al., 2018; Melero Ventola et al., 2020) and enhanced awareness of GD were associated with successful outcomes in terms of a reduced urge to gamble and GD treatment compliance (e.g., Castren et al., 2013; Jara-Rizzo et al., 2019).

The element *Perception* include opportunities to experience, test and adjust newly acquired understandings. For example, exposure to high-risk situations or distinctive emotional vulnerabilities during treatment sessions may offer opportunities to reevaluate and adjust one’s understanding of trigger traits (Pasche et al., 2013; e.g., Bouchard et al., 2017; van der Tempel et al., 2020). Additionally, testing and practicing coping strategies can provide opportunities to gain personal experiences with one’s coping strategies, and tailor the strategies accordingly (e.g., Grant et al., 2011; Boughton et al., 2016; Melero Ventola et al., 2020).

The third element, *Utilization*, involve possibilities to integrate new understandings and strategies to cope with triggering emotions, thoughts or experiences into real-life situations. Successful utilization was described as processes in which new coping strategies became embodied and integrated as an individual’s default responses to triggering emotions, thoughts, or situations. The participants from the study of van der Tempel et al. (2020) suggested that “more practice transferring skills from group sessions to home would have been valuable.” Further examples from the included studies are building traits such as abstinence self-efficacy (Gomes and Pascual-Leone, 2015) or confidence (Jackson et al., 2013), and daily practicing (Melero Ventola et al., 2020) or adherence to positive change (de Lisle et al., 2011).

### 3.4 Summary of key findings

**Figure 3:**
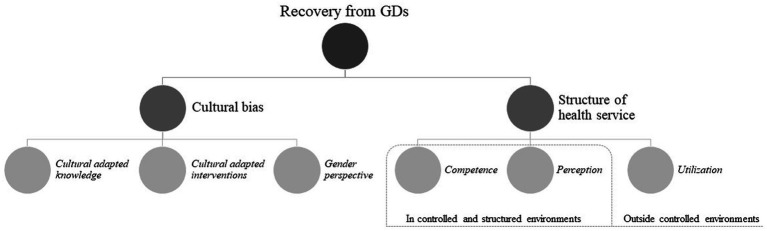
Key context variables for GD recovery.

First, the findings show that studies on GD interventions can be characterized by biases related to region, ethnicity, and gender perspectives (Cultural biases). Second, three elements have been identified in avenues to recovery (Competence, Perception, and Utilization). The key findings and relation between them are outlined in Figure 3.

## 4 Discussion

The aims of the present scoping review were to map the level of evidence and explore how contextual factors influence the provision and outcomes of interventions targeting emotional regulation or impulsivity in people with GDs.

The research on GD treatment is in the early stages of development, with most studies in the early phases of the five-phase model (Robey and Schultz, 1998). That is, establishing if different treatments influence outcomes and preparing for clinical trials. We propose an exploration of the deserved patterns and trends, and future research with focus on, such as different kinds of gambling, different settings, and cultural variations, for the field to move forward. On the other hand, one might argue that context is a central part of the disorder and, thus, part of the solution or treatment. In this case, one would not wait until phase IV to explore these variables but include them as covariates in early experimental studies, recommending multisite international collaboration.

The two key findings—Cultural biases and Three elements in avenues of recovery—will be discussed in the following sections.

### 4.1 Cultural biases in knowledge production—implications for practice

As pointed out by De Munck and Bennardo (2019), *culture* is a term used by many people and in varying ways. Anthropologist Clifford Geertz famously claimed that “man is an animal suspended in webs of significance he himself has spun” (Geertz, 1973). In line with Geertz, we refer to culture as a shared set of thoughts, meanings, norms, attitudes, values, and myths (see also Abbott et al., 2018).

A key finding from the present review is a lack of studies examining non-Western societies and specific cultural subgroups (e.g., ethnicity, religion, and Indigenous peoples). As pointed out by the researchers in the South African study (Pasche et al., 2013)—and still valid today—there is a lack of research on the effectiveness of treatment for GDs in low- and middle-income countries. This bias is unfortunate because research suggested that attitudes toward gambling and treatment can vary considerably by ethnicity and cultural traditions (Williams et al., 2012; Breen and Gainsbury, 2013; Clark, 2013; Kim et al., 2016; Abbott et al., 2018).

Another finding is a lack of research examining the impact of gender in the intervention of gambling problems. Although 25 out of 34 studies included both males and females in the sample, most did not explicitly address gender differences in the analysis. Overall, the gender perspective was the most present in the studies that exclusively used female samples. For example, Piquette and Norman (2013) claimed that most of the research in Western cultures has focused on the situation of American male gamblers and that there is a need for more research on intervention mechanisms that could support female problem gamblers. Similarly, van der Tempel et al. (2020) argued that gender often influences the trajectory of a GD, as several previous studies have also found (e.g., Baxter et al., 2016; Hing et al., 2016; Carneiro et al., 2020; Håkansson and Widinghoff, 2020). In line with these two studies, we argue that future research should integrate a gender perspective to a greater extent. This would allow for more insights into gender-related experiences of gambling, treatment, and GD, as well as how gender intersects with other factors (such as age, social class, and gambling type).

Health care policies and clinical practice are grounded on knowledge about the challenges they aim to solve and characteristics of the population they intend to serve (Brown and Ashford, 2019). The observed cultural bias in gambling research is most likely influenced by a complex interplay of factors. The dominance of English in academic publishing (Politzer-Ahles et al., 2020) and global research funding disparities (Hilbrecht et al., 2020) may play a pivotal role. Variations in cultural norms and gambling regulations across regions may also contribute. For instance, in areas with strict gambling laws, like many Middle Eastern countries, conducting gambling research is likely less feasible (Aldhehayan and Tamvada, 2023; Zeng et al., 2023). Additionally, in less affluent countries, pressing issues like poverty and inadequate healthcare infrastructure are likely to take precedence. A limited or biased knowledge base may have generative potential for provision and, therefore, outcomes of GD interventions, especially for populations for which there is a lack of knowledge about them (Grant and Chamberlain, 2023). To address this bias, a multifaceted approach involving inclusive funding, diverse publication channels, and international collaborations is essential (Hilbrecht et al., 2020). Such efforts could foster a more balanced understanding of GDs. The findings of the present review clearly suggest that there is a need for more attention to the role of cultural factors and gender in future gambling research.

### 4.2 Inside- or outside-controlled environments—implications for recovery from GD

Change-promoting interventions mostly consist of several phases or steps that may produce the intended outcomes (Pawson et al., 2004). Our findings imply that understanding the traits and behaviors that contribute to GD and having knowledge and experience with resilient coping strategies are important steps for navigating the complex processes of change. Furthermore, an equally important phase of the transformation process is gaining awareness and understanding of the personal triggers related to emotions, thoughts, or situations. These findings are in line with previous research on recovery from GD (see, e.g., Cornil et al., 2018; Oakes et al., 2020; Pickering et al., 2020). Individuals’ reactions to emotions are situational, especially in pressured circumstances, such as unexpected trigger situations (Gross, 2013; Tenenbaum et al., 2013). Trigger situations may impair cognitive function, hence influencing an individual’s capacity to regulate emotions and utilize newly gained knowledge and experiences with resilient coping strategies (Oakes et al., 2020). Emotional regulation includes strategies to modify or replace an affective state and efforts taken to identify the underlying cause of the affective state (Gross, 2013). The understanding of emotions arising in trigger situations affects the reactions to the emotion and, consequently, the actions taken to cope in such circumstances (Tenenbaum et al., 2013). Emotional reactions are influenced by both psychological (e.g., utilization of resilient coping strategies) and contextual (e.g., social support) conditions and vary depending on how triggering the specific situation is (Tenenbaum et al., 2013). In line with this, experiences with successful coping in situations that previously have triggered an unpleasant affective state may change the meaning the individual gives to similar situations (e.g., *unmanageable*). The new understanding of the situation (e.g., *manageable*) may facilitate or enable the individual’s ability to cope with similar challenges in the future (Gross, 2013), such as gambling-related trigger situations.

Different interventions are intended to initiate the features and processes described in two of the three elements. *Competence* contains interventions aiming to increase an individual’s insight, knowledge, and awareness (e.g., psychoeducation, cognitive therapy, conversational group therapy, etc.). *Perception* comprises interventions intending to enable opportunities to practice new strategies or enhance experience with emotions, thoughts, or situations (e.g., exposure therapy, virtual reality, body-based therapy, etc.). However, it seems that understanding these traits and behaviors and having knowledge of coping strategies alone may not be enough to successfully implement these strategies in the face of unexpected triggers in real-life situations. As the findings reveal, for GD patients to effectively use their knowledge and awareness, they need opportunities that enable the process where newly learned coping strategies are transformed and integrated as the standard response. Such opportunities should be formalized and integrated as services in the health care system.

The features and processes summarized earlier in *Utilization* are characterized by unplanned or unexpected episodes and situations where the newly gained insight or strategies are put to the test. In considering the prevailing treatment in conventional health care systems, interventions targeting traits embedded in *Competence* and *Perception* are planned according to a specific structure and progress in controlled environments (i.e., therapeutic setting, treatment facility, etc.). In contrast, features and processes covered by *Utilization* are not planned and occur in outside-controlled environments (i.e., in-between therapeutic sessions or after discharge from treatment). This points to a shortcoming of the formal health care system, representing a tendency that may be especially challenging for people recovering from disorders partly triggered by traits or activities that are conventional in most societies, such as gambling. Indeed, gambling is a legal activity in most countries worldwide, and the majority of players gamble with recreational motives (Calado and Griffiths, 2016; Health Survey for England 2018, 2019; Pallesen et al., 2020). Therefore, people who have experienced problems with gambling and have recovered from a GD are dependent on resilient strategies when facing unexpected trigger situations in their everyday lives.

Interventions that promote the features described in the elements *Competence* and *Perception* are well established within the formal health care system (see, e.g., Cowlishaw et al., 2012; Abbott, 2019; Bodor et al., 2021). However, treatment success extends beyond a successful outcome at the time of discharge from formal treatment. More specifically, treatment success depends not only on positive results within the treatment context, but also on successful outcomes outside of it. Our findings suggest that this requires insights and experiences gained in the treatment facility or therapeutic setting (i.e., in controlled and structured environments) become internalized and applied in real-life situations outside of the treatment context (i.e., outside-controlled environments). In line with this, we argue that these finding have structural and practical implications for stakeholders that are engaged in with GD services. Interventions promoting the processes described in *Utilization* may further facilitate avenues for recovery from GD, hence representing an important area for further research and a valuable contribution to the already established interventions in the formal health care system.

### 4.3 Limitations

Some limitations must be taken into account when considering the findings from the present study. First, this scoping review has applied an explorative approach in designing the search strategy and the analysis of the search results. However, the search strategy was informed by the findings from the preliminary search for treatment studies of prevailing GD interventions. Additionally, the search strategy and summary of the search results were driven by the intent to provide insights with relevance to today’s status in the practice field. Second, an alternative selection of databases, a different selection and combination of text words or studies written in other languages than English would generate other search results. Also, the first screening phase (titles) was primarily conducted by one author that evaluated all titles in the search result, while two authors evaluated 10% each of the titles. This entails a risk that eligible studies were not identified during the systematic search or excluded during the early screening process. However, the objective of scoping reviews is not to provide a complete overview of the existing evidence (Arksey and O'Malley, 2005; Tricco et al., 2018; Lockwood et al., 2019), and missing some studies for inclusion is the rule rather than the exception. Finally, the quality of the research design and data basis of studies were not assessed and, therefore, not considered in the analyses of the final included references. However, as shown in the discussion, the findings from this review are in line with established theories relevant to GD-related factors, as well as previous research findings with relevance to the aims of the current review.

## 5 Conclusion

The research field on GD treatment is in its infancy, with outcome studies mostly in the early phases of knowledge development. Findings from the current review of studies on treatment for GDs have structural and practical implications for various stakeholders that are engaged with GD research and services to promote recovery from GD. The review has uncovered context variables that must be taken into consideration when designing further research. First, there is a need to consider cultural biases and report on such variables (e.g., ethnicity and gender) to a greater extent. This may enhance knowledge about cultural variations and contribute to developing the availability of interventions adapted to specific minorities within the GD population. Second, health care services should include elements that enhance the competence related to GD and recovery from GD. Health care services should also provide the possibilities to practice newly gained strategies to cope. Finally, health care services should facilitate arenas that enable people in recovery from GDs to transform coping strategies into standard resilient responses when offering novel treatments.

## Data availability statement

The original contributions presented in the study are included in the article/Supplementary material; further inquiries can be directed to the corresponding author.

## Author contributions

DJ, SO, and EA contributed to conceptualization, planning the search strategy, screening and analyzing search results, and writing the manuscript. DJ conducted the systematic search and administrated the collaboration between the authors. All authors contributed to the article and approved the submitted version.

## Footnotes

^1^The registration was automatically rejected with the following reason: “To enable PROSPERO to focus on COVID-19 registrations during the 2020 pandemic, this registration record was automatically rejected because it did not meet the acceptance criteria.” Therefore, the record could not be edited and updated consecutively in PROSPERO, and changes made during the review process are thoroughly described throughout the section 2.

^2^Abbott et al. (2018, p. 72) defined the term as follows: “Indigenous Peoples refers to those who have occupied lands and territories before the arrival of settler societies.”
