# Supplementary material for: The role of contextual factors in avenues to recover from gambling disorder: a scoping review
Source: Front Psychol. 2024 Feb 12;15:1247152. doi: 10.3389/fpsyg.2024.1247152 (PMC10894926; doi:10.3389/fpsyg.2024.1247152)
Supplement: Supplementary file 1 [file Table_1.docx]

**Supplementary Table 1. Characteristics of the included studies**

| Author(s) (year) | Country | | | Aim | Study design | Target population | N | Setting | Intervention |
| --- | --- | --- | --- | --- | --- | --- | --- | --- | --- |
| Bouchard et al. (2017) | Canada | | | To document the potential of a minimal use of VR in CBT | Experiment, quantitative | People (mixed-gender, ethnic diverse) with GD | 34^^[[1]](#footnote-1)^^ | Laboratory experiment | VR gambling-exposure with participants recruited from GD treatment |
| Boughton et al. (2016) | Canada | | | To explore the viability and effectiveness of an internet-based intervention | Interventional, pre-post, Mixed Methods | Women with PG. ethnicity NI. | 11 | Digital intervention | Group-based webinar intervention combined with individual workbook-assignments between sessions |
| Castren et al. (2013) | Finland | | | To explore the impacts of Internet-based therapy | Interventional, follow-up, quantitative | People with gambling-related problems. Mixed gender, ethnicity NI. | 471 | Internet-based intervention | Cognitive therapy-based self-help program with telephone therapist support |
| Christensen et al. (2013) | Australia | | | To investigate the effectiveness of a brief DBT program | Interventional, pre-post, quantitative | People with PG and co-morbid disorders, described as treatment resistant. Mixed gender, ethnic diverse. | 24 | Outpatient specialised treatment service | Time-limited (9 weeks) Dialectical Behaviour Therapy provided by specialist problem gambling services |
| de Lisle et al. (2011) | Australia | | | To investigate the utility of mindfulness-based intervention | Case study with intervention, qualitative | Woman with PG. Ethnicity NI. | 1 | Weekly individual mindfulness-sessions with therapist | Time-limited (8 weeks) manualized mindfulness-based cognitive therapy to practice mindfulness skills and dealing with negative thought/feeling |
| Giroux et al. (2013) | Canada | | | To investigate the ability of repeated exposure to a virtual gambling environment to modify the urge to gamble and perceived self-efficacy | Experiment, qualitative | People with PG who wish to control or decrease their gambling activity. Mixed gender, ethnicity NI. | 10 | Laboratory experiment | VR exposure to a virtual gambling environment with video lottery players who wished to control or decrease their gambling habits |
| Gomes and Pascual-Leone (2015) | Canada | | | To examine how recovery resources and psychosocial stressors, predict positive treatment outcomes | Observational, qualitative | People with PG, recruited from treatment. Mixed gender, ethnic diverse. | 50 | Treatment for problem gambling | High levels of *Recovery resources* (social support, abstinence self-efficacy, readiness for change, motivation for change) |
| Granero et al. (2020) | Spain | | | To estimate the response trajectories of gambling severity after a CBT program | Exploratory, follow-up, quantitative | Young men (mean 29.7 years) with GD. Ethnicity NI. | 192 | Groupe-based outpatient GD treatment | Time-limited group program with 16 weekly 90-min sessions of Cognitive behavioural therapy (CBT) |
| Grant et al. (2010) | USA | | | To examine the safety and efficacy of pharmacological treatment with Memantine | Interventional, quantitative | People with GD. Mixed gender, ethnicity NI. | 29 | Medical intervention | Time-limited (10 weeks) treatment with Memantine |
| Grant et al. (2011) | USA | | | To examine whether the benefits of a brief intervention were maintained for at least 6 months | Interventional, quantitative | People with GD. Mixed gender, 97% white. | 44 | Individual outpatient GD treatment | Time-limited (8 sessions) of imaginal desensitization plus motivational interviewing (IDMI), a manualised treatment containing psychoeducation, motivational enchantment, coping strategies (cognitive and behavioural), trigger exposure, relaxation training and relapse prevention. |
| Jackson et al. (2013) | Australia | | | To examining the feasibility of whether leisure substitution may be an appropriate adjunct to individual counselling for the treatment of problem gambling | Evaluation, Mixed Methods | People in counselling for PG. Mixed gender, ethnicity NI. | 30 | Group-based program provided by clinicians and volunteers (support persons) | The Re(Making) Meaning project provided a structured re-engagement program activities designed to build teamwork and confidence in participants (e.g., bar-b-que, meditation, singing, learning computer skills, communication workshops, goal setting skills) |
| Jara-Rizzo et al. (2019) | Spain (Granada) | | | To estimate the predictive value of impulsivity for treatment dropout; and to test if confounders further predict the degree of compliance with therapeutic tasks and recommendations | Exploratory, follow-up, quantitative | People in group therapy for PG. Mixed gender, ethnicity NI. | 66 | Inpatient GD treatment | Long-term (about 2 years) mutual help group treatment complemented with professional supervision and individual cognitive-behavioural therapy |
| Ledgerwood et al. (2020) | Canada | | | To explore factors related to gambling-related cognitive distortions over the course of treatment and its predictive value for treatment outcome | Exploratory, pre-post, quantitative | People in inpatient treatment for GD. Mixed gender, ethnicity NI. | 125 | Residential GD treatment | Time-limited (21 days) higher level (residential) gambling care (content not further described) |
| Mallorqui-Bague et al. (2018) | Spain | | | To determine the predictive power of executive function and trait impulsivity | Exploratory, longitudinal, qualitative | Men in outpatient treatment for GD. Ethnicity NI. | 144 | Specialised outpatient GD service | Time-limited (16 weekly 90-minuts group sessions) manualised cognitive-behavioural treatment (CBT) led by a clinical psychologist and co-therapist. Group sessions included topics to foster GD preventive strategies. |
| Melero Ventola et al. (2020) | Spain | | | To evaluate the effect of a mindfulness-based cognitive group therapy on gambling-related craving | Interventional, follow-up, quantitative | Men in group therapy for PG. Ethnicity NI. | 33 | Manualised group intervention | Time-limited (8 weeks) mindfulness-based cognitive therapy (MBCT) training to reduce gambling-related craving. MBCT consist of psychoeducation, identifying cognitive distortions, cognitive reappraisal, mindfulness exercises and imaginal exposure. |
| Mutschler et al. (2010) | Germany/ Switzerland | | | To explore the potential of pharmacological treatment with disulfiram to reduce craving | Case study, qualitative | Men with PG. Ethnicity NI. | 1 | Medical intervention | Treatment with Disulfiram provided by a physician three times per week for 12 months, including high frequency of short-term individual contacts, reinforcement of abstinence and development for alternative coping skills |
| Nastally and Dixon (2012) | USA | | | To evaluate the effect of brief Acceptance commitment therapy on subjective probabilities of winning | Experiment, pre-post, quantitative | People with PG. Mixed gender, ethnicity NI. | 3 | Laboratory experiment | Acceptance commitment therapy delivered via a Microsoft PowerPoint presentation within a brief experimental session to increase acceptance, defusion, self as context, contact with the present moment, values, and committed action in participants |
| Nuske and Hing (2013) | Australia | | | To provide a narrative analysis of the stories of problem gambling recovery | Cross-sectional, interview, qualitative | People with PG. Mixed gender, ethnicity NI. | 10 | Formal and informal health care services | Experiences with help-seeking pathways and avenues to recovery from GD |
| Parhami et al. (2012) | USA | | | To examine the effectiveness of a culturally adapted telephone intervention | Interventional, follow-up, quantitative | Asian Americans with PG. Mixed gender. | 8 | Digital community-based intervention | Culturally adapted (Asian Americans) gambling intervention consisted of six 1-hr long telephone-delivered sessions (psychoeducation, self-assessment tools, relapse prevention techniques) based on motivational enhancement and treatment of cognitive distortions |
| Pasche et al. (2013) | South Africa | | | To evaluate the therapy program IDMI to treat patients with PG | Evaluation, quantitative | South African people with PG. Mixed gender. | 128 | Outpatient GD treatment with trained therapists | Manualised time-limited (weekly sessions for 6 (7) weeks) intervention with imaginal desensitization plus  motivational interviewing (IDMI), imaginal exposure and homework. IDMI provide participants with specific skills (GD psychoeducation, trigger and financial planning, relapse prevention) which they practice between sessions |
| Piquette and Norman (2013) | Canada | | | To explore experiences of all-female group counselling for problem gambling | Follow-up interview study, qualitative | Caucasian Canadian women in counselling for PG. | 4 | Women-only treatment group offered through a provincial health agency | A female-only intervention group (weekly 3-hour sessions for 12 weeks) for problem gambling facilitated by a trained counsellor. Sessions were devoted to psychoeducational (first 90 min) and open-group process (last 90 min). |
| Rodda et al. (2017) | Australia | | | To evaluating the effectiveness of a single session of web-based counselling | Evaluation, quantitative | People with PG. Mixed gender, ethnicity NI. | 229 | Online immediate counselling | A free, 24/7 telephone service with counselling, information and support to anyone affected by problem gambling |
| Rossini-Dib et al. (2015) | Brazil | | | To measure treatment outcomes and identify factors that could be associated with clinical recovery | Exploratory, pre-post, quantitative | People in outpatient treatment for PG. Mixed gender, ethnic diverse. | 113 | Outpatient group-treatment for GD | Time-limited (4 months) psycho-education group, psychiatric treatment and cognitive behavioural group therapy (content not further described) |
| Shead et al. (2020) | Canada | | | To explore the relationship between gambling, mindfulness and delay discount, and the potential of meditation to change craving | Exploratory, observational, quantitative | People with gambling-related problems. Mixed gender, ethnicity NI | 59 | Digital intervention | Short-term (10 min daily listening-sessions for 1 week) audio-guided mindfulness-based meditation exercises |
| Smith et al. (2016) | Australia | | | To extend findings from a randomised trial to evaluate the differential efficacy of CT versus ET | Cross-sectional, interview, qualitative | People in treatment for PG. Mixed gender, ethnicity NI. | 8 | Outpatient GD treatment | Cognitive and behavioural therapy, focussing on clients’ urge to gamble using exposure therapy (ET).  Cognitive therapy (CT) focussing on restructure clients erroneous gambling belief. |
| Smith et al. (2018) | Australia | | | To explore whether treatment outcomes derived from randomized controlled trials (RCTs) still hold when applied to patients seen in routine practice | Comparative, quantitative | People in treatment for PG. Mixed gender, ethnicity NI. | 320 | Outpatient GD treatment | Cognitive and behavioural therapy, focussing on clients’ urge to gamble using exposure therapy (ET).  Cognitive therapy (CT) focussing on restructure clients erroneous gambling belief. |
| Stewart et al. (2016) | Canada | | | To assess the utility of a motivation-matched treatment | Case series, qualitative | Caucasian men with PG. | 6 | Individual therapist-led intervention | Manualised motivation-matched treatment (weekly session for 6 week) designed to address gambling motive (escape- or action gamblers) and maladaptive thinking patterns unique to each gambling motive subtype. |
| Syvertsen et al. (2020) | Norway | | | To explore experiences with a self-help group | Cross-sectional, interview, qualitative | People with PG. Mixed gender, ethnicity NI. | 9 | Self-help group | Weekly 2-hour self-help groups for problem gamblers or affected others, led by volunteers who are provided with guidelines (e.g. requiring confidentiality and members to aim for gambling abstinence) and plan for meetings (e.g. to exchange advice and experiences, and opportunities to invite guest lecturers). |
| Tarrega et al. (2015) | Spain | | | To evaluate the possible additional impact of a serious videogame in a CBT | Evaluation, pre-post, quantitative | Men in treatment for PG. 93.8% of participants originated from study country. | 16 | Psychologist-led outpatient group intervention | CBT intervention consisted of 16 group weekly sessions (psychoeducation, stimulus control, response prevention, cognitive restructuring, self-reinforcement, relapse prevention), and 10 weekly sessions of a serious video game (problem solving,  planning and self-control skills, relaxation skills) |
| van der Tempel et al. (2020) | Canada | | | To investigate the feasibility of group-based mindfulness-based intervention | Interventional, feasibility, quantitative | Help-seeking women with PG. Ethnicity NI. | 9 | Clinician-led group intervention for women | Manualised mindfulness-base group sessions (weekly for 10 weeks) focusing on gambling triggers and urges/actions leading to gambling, with between-session homework. |
| van Minnen et al. (2020) | The Netherlands | | | To gaining insight in the efficacy of a shortened versions of Eye movement desensitisation | Interventional, multiple baseline study, Mixed Methods | People in treatment for PG. Mixed gender, ethnic diverse. | 8 | Outpatient therapist-led intervention | Eye movement desensitization and reprocessing (EMDR) therapy (weekly sessions for 6 weeks) focusing on visualisation expositor (motivation, loss of control) and restructuring |
| Ward et al. (2018) | UK | | | To investigate the potential indications and adverse effects of using the opioid antagonist naltrexone to treat problem gamblers | Case series, qualitative | People with GD or PG. Mixed gender, ethnicity NI. | 10 | Medical intervention | Patients were prescribed an initial dose of 25 mg naltrexone per day for 3 days, and then 50 mg per day as the usual maintenance dose (offered through study for 8 weeks before prescription was handed over to respective general practitioner). |
| Zack et al. (2016) | Canada | | | To assess the effects of continuous theta burst stimulation on gambling reinforcement and related responses | Experiment, repeated measure, quantitative | Men with PG. Ethnicity NI. | 9 | Laboratory experiment | Weekly intervals with high frequency rTMS of the medial prefrontal cortex (3 epochs (15 10-pulse trains of 1-s duration) with 5-min intervals between) and continuous theta burst stimulation (cTBS; 3 epochs (900 pulses) with 5-min intervals between). |
| Zhuang et al. (2018) | Hong Kong | | | To examine the short- and long-term effect of a culturally attuned integrated CBI group treatment | Quasi-experiment, paired comparison, quantitative | Chinese men with PG living in Hong Kong. | 84 | Therapist-led group intervention | Time-limited (weekly sessions for 8 weeks) cognitive behavioural intervention focussing on motivation, gambling distortions and beliefs, urges and triggers to gamble. Cognitive and behavioural strategies were taught and practiced within and outside the group sessions. |
| N | | Mean: 68  Median: 27.5  Min/max: 1/471 | | | | | | | |
| Total: | | 2298 (female; minimum 594) | | | | | | | |
| Year of publication: | | Mean: 2016  Mode: 2013 | | | | | | | |
| Type of study: | | Quantitative: 23  17 Exploratory studies  5 Experimental studies  1 Comparative study  Qualitative: 8  4 Cross-sectional interview studies  4 Case study/case series  Mixed Methods: 3  1 Multiple baseline study with feedback from participants  1 Programme evaluation with feedback from participants  1 Pre-post study with feedback from participants | | | | | | | |
| Abbreviations: | | |  | |  |  |  |  |  |
| GD | | Gambling disorder | | |  |  |  |  |  |
| NI | | Not Identified | | |  |  |  |  |  |
| PG | | Problem gambling | | |  |  |  |  |  |

1. *Study 2* [↑](#footnote-ref-1)
