# Supplementary material for: The role of contextual factors in avenues to recover from gambling disorder: a scoping review
Source: Front Psychol. 2024 Feb 12;15:1247152. doi: 10.3389/fpsyg.2024.1247152 (PMC10894926; doi:10.3389/fpsyg.2024.1247152)
Supplement: Supplementary file 2 [file Data_Sheet_1.DOCX]

**SI 1. Serac strategy**

**Complete search strategy**

| # 6 | # 3 AND # 4 AND # 5 |
| --- | --- |
| # 5 | urge* OR emotion* OR affect* OR valenc* OR reappraice* OR toleranc* OR impulsiv* OR desire* OR crave* OR attent* OR aware* OR accept* OR inhibit* OR regulat* OR control* OR decision* OR avoid* OR seek* OR reward* OR novelty* OR action* |
| # 4 | therap* OR intervention* OR psycother* OR counsel* OR treatment* OR service* OR self-help OR self-direct* OR “user driven” OR peer* OR NGO OR "health care" OR "social service" |
| # 3 | # 1 AND # 2 |
| # 2 | problem* OR patholog* OR disorder* OR addict* |
| # 1 | gambl* |

**Example of search from Ovid**

| 1. | gambling/ |
| --- | --- |
| 2. | exp Addiction/ |
| 3. | 1 and 2 |
| 4. | exp treatment/ |
| 5. | 3 and 4 |
| 6. | gambl*.ab. |
| 7. | (problem* or patholog* or disorder* or addict*).ab. |
| 8. | 6 and 7 |
| 9. | (urge* or emotion* or affect* or valenc* or reappraice* or toleranc* or impulsiv* or desire* or crave* or attent* or aware* or accept* or inhibit* or regulat* or control* or decision* or avoid* or seek* or reward* or novelty* or action*).ab. |
| 10. | (therap* or intervention* or psycother* or counsel* or treatment* or service* or self-help or self-direct* or "user driven" or peer* or NGO or "health care" or "social service").ab. |
| 11. | 8 and 9 and 10 |
| 12. | 5 or 11 |
